# Hsa_circ_0023642 promotes proliferation, invasion, and migration of gastric cancer by sponging microRNA‐223

**DOI:** 10.1002/jcla.23428

**Published:** 2020-06-19

**Authors:** Yi Zhang, Lei Xia, Jing Wu, Xinyu Xu, Gang Li

**Affiliations:** ^1^ Department of Pathology Jiangsu Cancer Hospital Jiangsu Institute of Cancer Research The Affiliated Cancer Hospital of Nanjing Medical University Nanjing China; ^2^ Department of Radiation Oncology Jiangsu Cancer Hospital Jiangsu Institute of Cancer Research The Affiliated Cancer Hospital of Nanjing Medical University Nanjing China; ^3^ Department of General surgery Jiangsu Cancer Hospital Jiangsu Institute of Cancer Research The Affiliated Cancer Hospital of Nanjing Medical University Nanjing China

**Keywords:** gastric cancer, hsa_circ_0023642, invasion, migration, proliferation

## Abstract

**Background:**

Circular RNAs (circRNAs) have a closed‐loop structure and are associated with various cellular biological processes, including carcinogenesis and cancer development. However, our knowledge of circRNAs in gastric cancer (GC) remains limited. Thus, this study aimed to investigate the role and underlying molecular mechanisms of hsa_circ_0023642 in GC.

**Materials and methods:**

Bioinformatic analysis revealed that hsa_circ_0023642 was upregulated in GC. Chromogenic in situ hybridization (CISH) chips was used to explore the relationship between the expression of hsa_circ_0023642 and the malignant degree, clinical stage, and prognosis of patients with GC. The role of hsa_circ_0023642 in GC was assessed in vitro, and biotin‐coupled RNA pull‐down was conducted to evaluate the interaction in between hsa_circ_0023642 and miR‐223.

**Results:**

The current study showed that hsa_circ_0023642 was upregulated in GC and presented a high positive correlation with the malignant progression of GC. In addition, in vitro experiments showed that the silencing of hsa_circ_0023642 in GC cell lines MKN‐45 and SGC‐7901 significantly reduced the proliferation, invasion, and migration of GC cells. We confirmed that hsa_circ_0023642 could serve as a sponge of miR‐223, subsequently promoting GC progression.

**Conclusion:**

This study shows that the upregulation of hsa_circ_0023642 affects the malignant progression, clinical stage, and prognosis of GC which provides a new molecular target for the therapy of GC.

## 1 INTRODUCTION

Gastric cancer (GC) is a gastrointestinal cancer with high incidence, especially in China and Japan.^1^ From 2009 to 2011, 679 100 patients in China were diagnosed with GC, and 498 000 of them died.^2^ Given that the molecular and genetic basis of GC has not been clearly elucidated, most patients with GC are diagnosed with advanced disease and have poor prognosis.^3^, ^4^ Therefore, identifying new biomarkers and therapeutic targets to improve GC diagnosis and treatment is critical.

Circular RNA (circRNA) represents a new class of conserved endogenous RNA; unlike linear RNA produced by normative splicing, circRNA may be produced by a process called reverse splicing.^5^, ^6^ This non‐canonical splicing can produce three types of circRNAs, namely exon circRNA (circRNA), circular intron RNA, and exon‐intron loop RNA. CircRNA can play an important role in regulating cancer progression, such as cancer metastasis, apoptosis, and invasion, by acting as a miRNA sponge and forming a circRNA‐miRNA axis.^7^, ^8^ Unlike other non‐coding RNAs, which are stable due to their loop structure, circRNA is present in plasma, platelets, and exosomes and is therefore a potential biomarker for cancer diagnosis and treatment.^9^, ^10^ Thus, further elucidation of the function of dysregulated circRNA in cancer will help to improve cancer diagnosis and treatment.

This study was designed to explore the relationship between the malignant progression of GC and a new circRNA named hsa_circ_0023642, whose corresponding target gene is called UVARG. In this study, we examined the expression levels of hsa_circ_0023642 in GC tissues, analyzed the correlation between their expression levels and malignant stress and clinical features, and explored the clinical significance of hsa_circ_0023642. We also studied the expression of hsa_circ_0023642 in vitro cell lines through cell proliferation and cell migration assays. Predictions and annotations of hsa_circ_0023642 molecular function with miRNA or mRNA were performed on relevant databases.

## 2 MATERIALS AND METHODS

### 2.1 Microarray analysis

The gene expression profile matrix files from GSE78092 based on GPL21485 platform, including 3 pairs GC samples were downloaded from the Gene Expression Omnibus (GEO) database (https://www.ncbi.nlm.nih.gov/gds). Gene expression data for the matrix file was subjected to normalized signal intensity. The average RNA expression was taken when duplicate data were found, and low‐abundance RNA‐sequencing data were removed.

The scale method of the “limma” R package (Version 3.42.2; https://bioconductor.org/packages/release/bioc/html/limma.html) was used to normalize the data. The heatmap was generated using the “pheatmap” R package (Version: 1.0.12; https://cran.r‐project.org/web/packages/pheatmap/index.html). Above statistical analyses of this study were conducted using R software (Version 3.6.2).

### 2.2 Patient and clinical tissue samples

We collected GC specimens from patients who underwent surgery from January 2015 to October 2018 in the Jiangsu Cancer Hospital. A total of 60 GC tissues and corresponding normal gastric tissue samples were obtained. The median age of the samples collected was 56.5 years, and neither radiotherapy nor chemotherapy was received before surgery. For the samples obtained, we accurately staged all tumors according to the International Cancer Alliance's Tumor‐Lymph Node Metastasis (TNM) Staging System (v.8; 2016). Age, gender, diameter, degree of differentiation, lymph node metastasis, TNM staging, and common clinical biomarker levels were analyzed. Prior to participating in the study, each participant signed a written informed consent form. This study was approved by the Medical Ethics Committee of Nanjing Medical University.

### 2.3 Chromogenic in situ hybridization

The collected cancer and adjacent tissues were embedded in wax blocks, and the wax pieces were frozen and sliced to a thickness of 5‐8 µm for use. In situ hybridization experiments were performed according to the procedure indicated in the kit, and probes for circRNA were prepared, covered and finally stained into pieces by DAB. According to the expression of staining, 60 samples were statistically scored for the expression level of circRNAs. The correlation between the expression level and the malignant progression, clinical analysis, and clinical prognosis of GC was analyzed.

### 2.4 Cell culture

Human GC cell lines MKN‐45, BGC‐823, MGC‐803, SGC‐7901, and the human gastric epithelial cell line GES‐1 were obtained from the Nanjing Medical University. We extracted RNA from four GC cell lines and the human gastric epithelial cell line GES‐1, tested the expression of hsa_circ_0023642 by RT‐PCR. Two GC cell lines with high expression were selected as the research object. We designed two siRNAs that could target the back‐splice junction of circ0023642. The expression level of circ0023642 was successfully decreased after siRNAs were transfected into MKN45 and SGC7901 cells, and si‐circ0023642#2 was chosen for further experiments due to its higher inhibitory efficiency.

### 2.5 Cell proliferation assays

CCK‐8 assay was performed according to the protocol provided by the manufacturer. We collected log phase cells, added adjusted cell suspension concentration to each well, and plated the cells to be tested to a density of 5000 cells/well. After incubation, CCK‐8 solution was added, and the culture was continued. When considerable color change occurred, the absorbance of each well was measured at an automatic microplate reader at OD 450 nm, and cell survival rate was calculated.

### 2.6 Cell migration assays

For migration assays, the drug‐treated cells were digested from a six‐well plate to adjust the cell density to 5 × 105/mL, 200 µL of which was inoculated into the upper chamber; the lower chamber was added to complete the medium for culture. After the cultured cells were fixed, washed, inverted, and air‐dried, 100 µL of the diluted crystal violet staining solution was added for staining. The cells were washed with ddH_2_0 to remove excess crystal violet and were then photographed. For each chamber, the number of cells was counted in four random fields, and the average was calculated.

### 2.7 Cell invasion assays

Matrigel was completely melted at 4°C and diluted with serum‐free basal medium. Approximately 50‐100 µL of Matrigel was then added per well to coat the Transwell chamber. The chamber was then placed in a 37°C incubator for 2‐3 hours. The drug‐treated cells were digested from a six‐well plate and inoculated in the upper chamber under the conditions for cell culture. After cell fixation and staining, crystal violet was washed off twice with ddH_2_0, and the cells were photographed. The cells on the underside of the membrane were counted to determine the number of migrated cells.

### 2.8 Prediction of miRNA targets

Interactions between circRNAs and miRNAs were predicted using Targetscan and starBase with the miRanda algorithm.

### 2.9 Biotin‐labeled pull‐down assay

A biotinylated circ0023642 probe and oligo probe (RiboBio) were synthesized and incubated with streptavidin magnetic beads (Life Technologies) at 25°C for 2 hours to generate probe‐coated beads. A total of 1 × 10^7^ GC cells were harvested, lysed, and sonicated. The cell lysates were incubated with probe‐coated beads at 4°C overnight. The beads were then washed with buffer, and the RNA complexes bound to the beads were eluted and extracted with a RNeasy Mini Kit (Qiagen). The abundance of circ0023642 and immunoprecipitated miRNAs was measured by qRT‐PCR.

### 2.10 RNA extraction and quantitative reverse‐transcription polymerase chain reaction

Total RNA was extracted from 4‐μm‐thick FFPE specimens by manual microdissection using RNeasy FFPE Kit (Qiagen). The complementary DNA (cDNA) synthesis was performed using PrimeScript RT Master Mix (RR036A) (TAKARA). The quantitative reverse‐transcriptase polymerase chain reaction (RT‐PCR) assays were performed using ViiA 7 Dx RT‐PCR System (Applied Biosystems) using PowerUp SYBR Green Master Mix (Applied Biosystems). The cycling conditions were as followed by 40 cycles of 95°C for 15 seconds and 60°C for 60 seconds. The relative expression of target genes was normalized against glyceraldehyde‐3‐phosphate dehydrogenase (GAPDH) using the 2^−ΔCT^ method.

### 2.11 Statistical analysis

Differences between tumor tissues and normal gastric tissues were analyzed by a paired *t* test, whereas categorical data were analyzed by chi‐square test. We assessed the diagnostic value by establishing a receiver operating characteristic curve. GraphPad Prism7.0 (Graph Pad Software) was used to plot all graphs. For all statistical results, *P* < .05 was considered statistically significant.

## 3 RESULTS

### 3.1 Expression of hsa_circ_0023642 is increased in GC tissues

**FIGURE 1:**
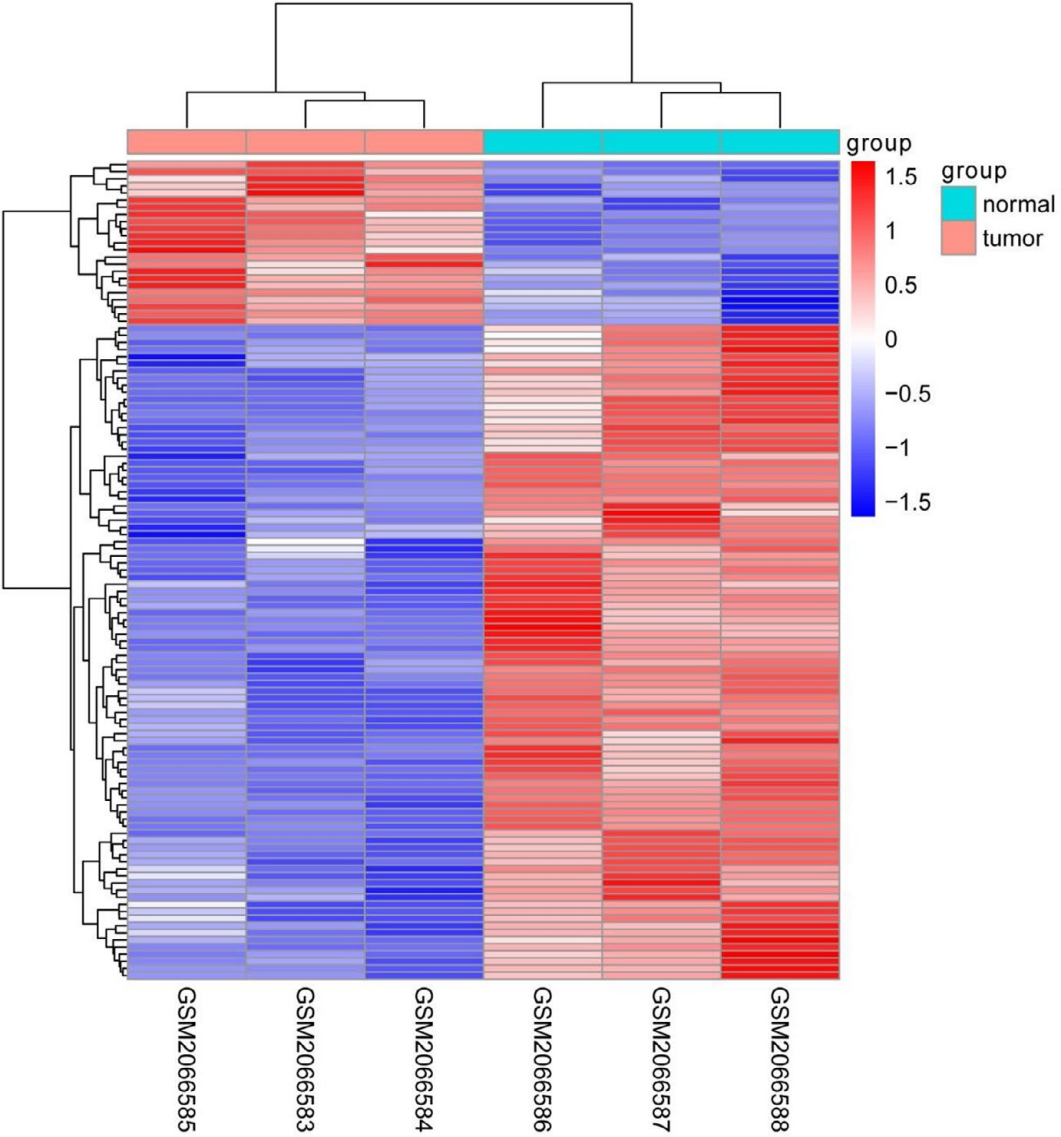
Hierarchical clustering, volcano plots, and scatter plots exhibited the differentially expressed circRNAs in gastric cancer tissues compared with paired non‐gastric cancer tissues

We used the multi‐dataset online analysis platform GEO dataset (GSE78092) to conduct an in‐depth screening of GC circRNA chips. In the early screening, using “limma” R package, there were 115 circRNAs identified that were statistical significantly differently expressed (|LogFC| > 1, *P* < .05) between normal tissues and tumor tissues, including 82 highly expressed and 23 lowly expressed circRNA were obtained. The expression abundance and difference of hsa_circ_0023642 in cancer tissues were the highest (Figure 1).

### 3.2 Hsa_circ_0023642 is correlated with the clinicopathological features and overall survival of patients with GC

**FIGURE 2:**
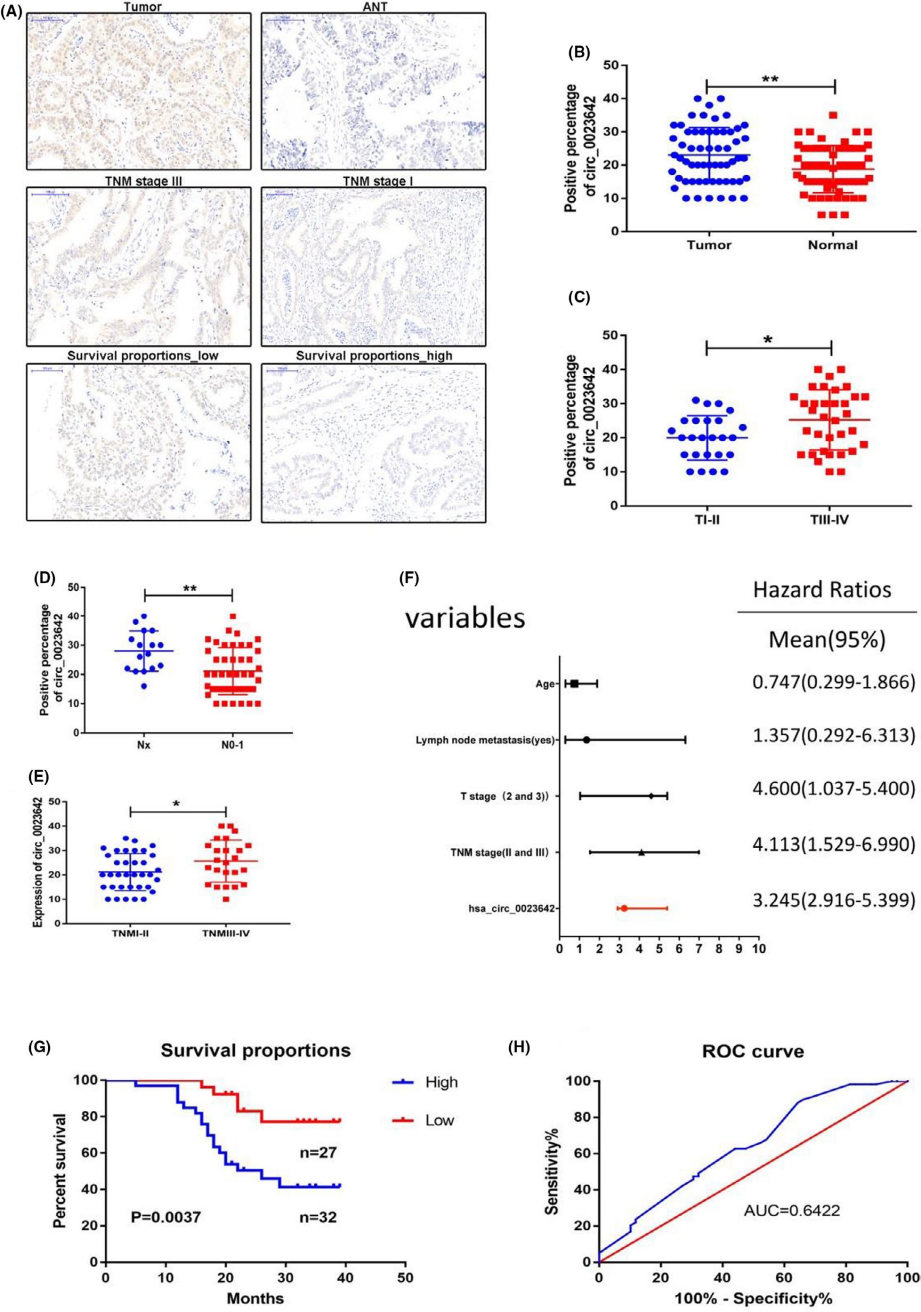
Relative hsa_circ_0023642 expression and its clinical significance in GC CISH chip. A, The expression of hsa_circ_0023642 in CISH chip: The upper shows the expression of hsa_circ_0023642 levels was significantly higher than ANT tissues; the middle shows the expression of hsa_circ_0023642 levels was significantly higher in severe TNM tumor; the lower shows high expression of hsa_circ_0023642 has a worse overall survival. B, The expression of hsa_circ_0023642 expression levels was significantly higher than that of adjacent noncancerous tissues. C, The expression of hsa_circ_0023642 expression levels was significantly higher in severe cases of primary tumor. D, The expression of hsa_circ_0023642 expression levels can predict the degree of lymph node metastasis. E, The expression levels can predict severe clinical stage. F, Hsa_circ_0023642 can be used as an independent predictor for patients with gastric cancer. G,The survival curve showed that patients with high expression of hsa_circ_0023642 had short survival time (*P* = .0037). H, Hsa_circ_0023642 is a sensitive indicator for the prognosis of gastric cancer patients, the AUC was 0.6422. AUC, area under the ROC curve; GC, gastric cancer; ROC, receiver operating characteristic; ns, not significant

Utilizing an GS tissue microarray (TMA) containing 60 paired GS tissues and their corresponding ANT tissues, we then measured the correlation of hsa_circ_0023642 expression with clinicopathological characteristics by in situ hybridization (ISH). The results indicated that the express level of hsa_circ_0023642 was significantly higher in GS tissues than in matched ANT tissues (Figure 2B). A univariate analysis showed that age, lymph node metastasis, T stage, TNM stage, and hsa_circ_0023642 expression level were significantly correlated with overall survival (OS) (Figure 2C‐E and Table 1). Subsequently, using these variables, the multivariate analyses indicated that the hsa_circ_0023642 expression level was an independent risk factor for OS (Figure 2F; Table 2). Furthermore, Kaplan‐Meier's survival curves showed that the patients with GS and higher hsa_circ_0023642 expression had poorer OS (Figure 2G‐H).

**TABLE 1:** Clinicopathological characteristics and expression of hsa_circ_0023642

Variables	Cases	Normal expression	Overexpression	*χ* ^2^	*P*‐value
Age (y)	59			0.5928	.4414
>60	33	12	21		
<60	26	7	19		
Gender	59			0.2151	.6428
Female	12	5	7		
Male	47	21	26		
Diameter	59			4.515	**.0336**
4	37	15	22		
4	22	14	8		
Differation	59			0.0006	.9803
Low/middle	50	22	28		
Well	9	4	5		
Tumor stage	59			4.468	**.0345**
T I‐II	25	15	10		
T III‐IV	34	13	21		
Lympatic metastasis	59			12.74	**.0004**
N0‐1	43	25	18		
Nx	16	3	13		
TNM stage	59			5.968	**.0146**
I‐II	35	20	15		
III‐IV	24	8	16		
Status	59			10.88	**.001**
Dead	23	6	17		
Alive	36	22	14		

Abbreviation: TNM, tumor node metastasis.

*P*‐value is 0.0391

**TABLE 2:** Univariate and multivariate Cox regression analyses of overall survival in 59 GS patient

Parameters	Univariate analysis			Multivariable analysis		
	HR	95% CI	*P* value	HR	95% CI	*P* value
Age (>60)	0.364	0.066‐2.006	.046	0.747	0.299‐1.866	.032
Gender (male)	2.569	1.60‐5.365	.554	－	－	－
Lymph node metastasis (positive)	2.366	0.432‐3.950	.021	1.357	0.292‐6.313	.017
T stage (2 and 3)	0.521	0.060‐1.507	.004	4.6	1.037‐5.400	.045
TNM stage (II and Ⅲ)	3.028	1.587‐7.525	.009	4.113	1.529‐6.990	.039
Histologic grade (M‐L and L)	0.747	0.137‐1.086	.737			
Hsa_circ_0023642 (high)	4.052	0.052‐6.105	.008	3.245	2.916‐5.399	.391

Abbreviation: TNM, tumor node metastasis.

*P* value is 0.0391.

### 3.3 Knockout of hsa_circ_0023642 suppresses the proliferation, invasion, and migration of GC cells

**FIGURE 3:**
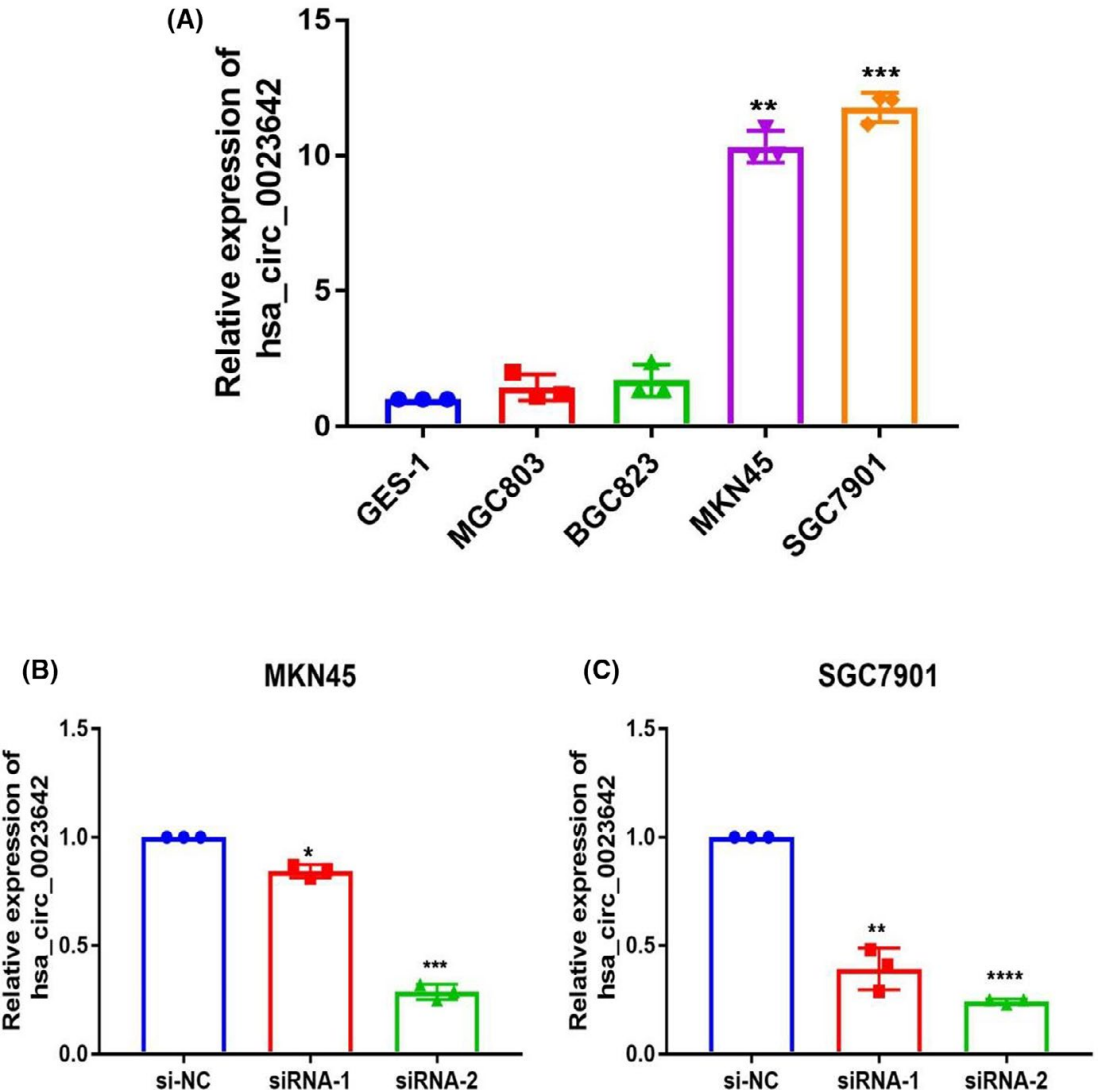
The expression of hsa_circ_0023642 in GC cell lines. A, The expression of hsa_circ_0023642 in gastric cells was higher than that in GES‐1 significantly. B, Knockdown of hsa_circ_0023642 was confirmed via qRT‐PCR, demonstrating the effective knockdown in MKN‐45 cells. C, Knockdown of hsa_circ_0023642 was confirmed via qRT‐PCR, demonstrating the effective knockdown in SGC‐7901 cells. *P < .05, **P < .01, ***P < .001. GC, gastric cancer; qRT‐PCR, quantitative real‐time polymerase chain reaction; si‐NC, negative control

**FIGURE 4:**
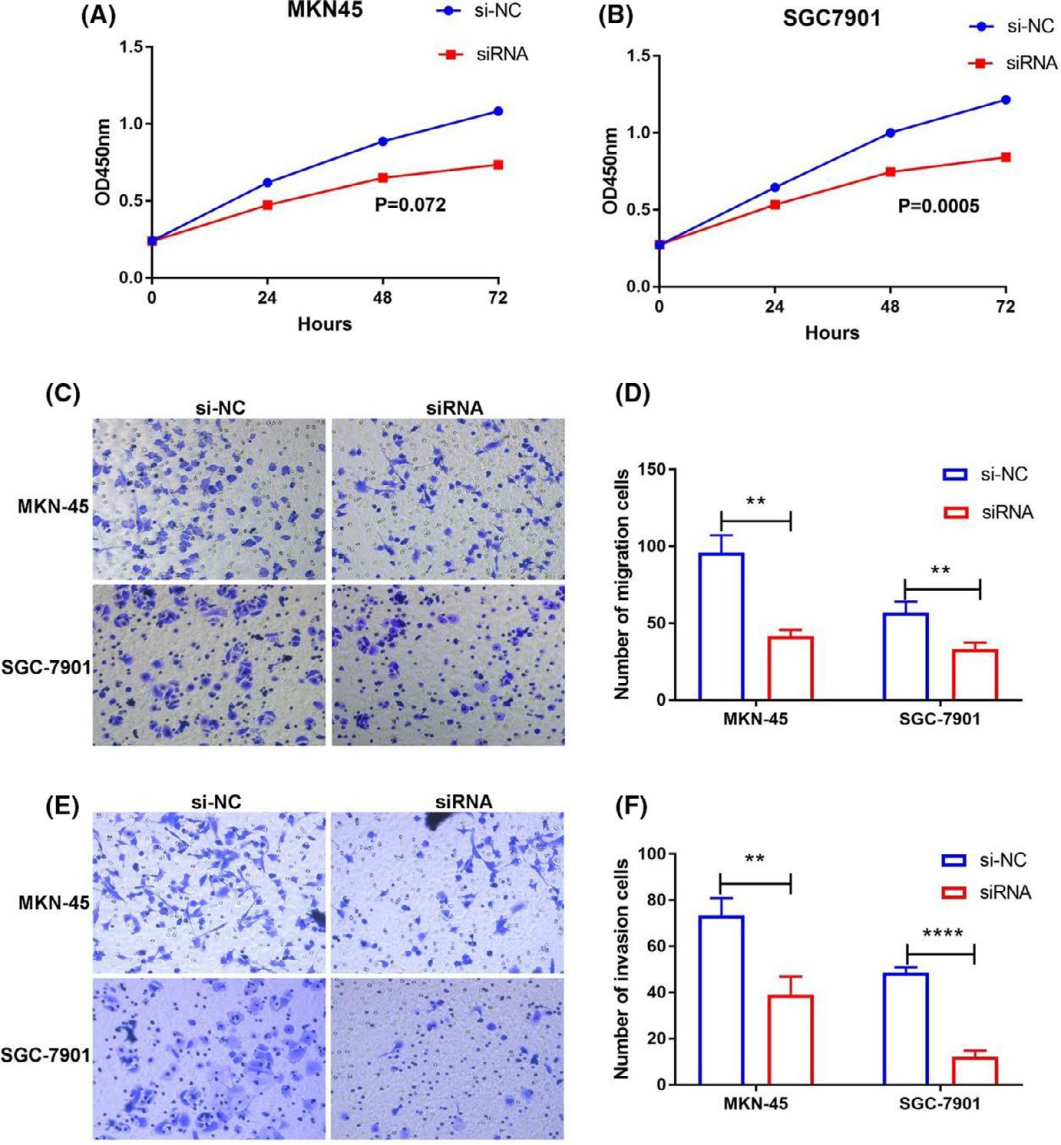
hsa_circ_0023642 promotes the growth, invasion and migration of GC cells in vitro. A, Knockdown of circ‐0023642 inhibited cell proliferation significantly in MKN‐45 cells. B, Knockdown of circ‐0023642 inhibited cell proliferation significantly in SGC‐7901 cells. C, MKN‐45 and SGC‐7901 cells infected with si‐hsa_circ_0023642 displayed significantly lower migration capacity compared with those infected with si‐NC. Scale: 40 × 10. D, Knockdown of hsa_circ_0023642 decreased MKN‐45 and SGC‐7901 cell migration significantly. E, MKN‐45 and SGC‐7901 cells infected with si‐hsa_circ_0023642 displayed significantly lower invasion capacity compared with those infected with si‐NC. Scale: 40 × 10. F, Knockdown of hsa_circ_0023642 decreased MKN‐45 and SGC‐7901 cell invasion significantly. **P < .01. GC, gastric cancer; si‐NC, negative control

To further investigate the function of hsa_circ_0023642, we tested its presence in GC cell lines (MKN‐45, MGC‐803, BGC‐823, SGC‐7901). We then selected cell lines with high expression levels of hsa_circ_0023642 to perform silencing experiments (Figure 3). CCK‐8 experiment was carried out to measure the proliferation of GC cell lines. The results showed that the downregulation of hsa_circ_0023642 can effectively prevent the proliferation of GC cell lines (Figure 4A‐B). We evaluated the effects of hsa_circ_0023642 on the migration and invasion behavior of GC cell lines and found that the transmembrane migration ability of GC cells infected with si‐hsa_circ_0023642 was lower than that of si‐NC–infected patients (Figure 4C‐D). GC cell invasion experiments showed that the silencing of hsa_circ_0023642 effectively inhibited the invasive ability of GC cell lines (Figure 4E‐F). These results indicate that hsa_circ_0023642 downregulation inhibits the proliferation, invasion, and migration of GC cells.

### 3.4 Hsa_circ_0023642 serves as miR‐223‐3p sponge in GC cells

**FIGURE 5:**
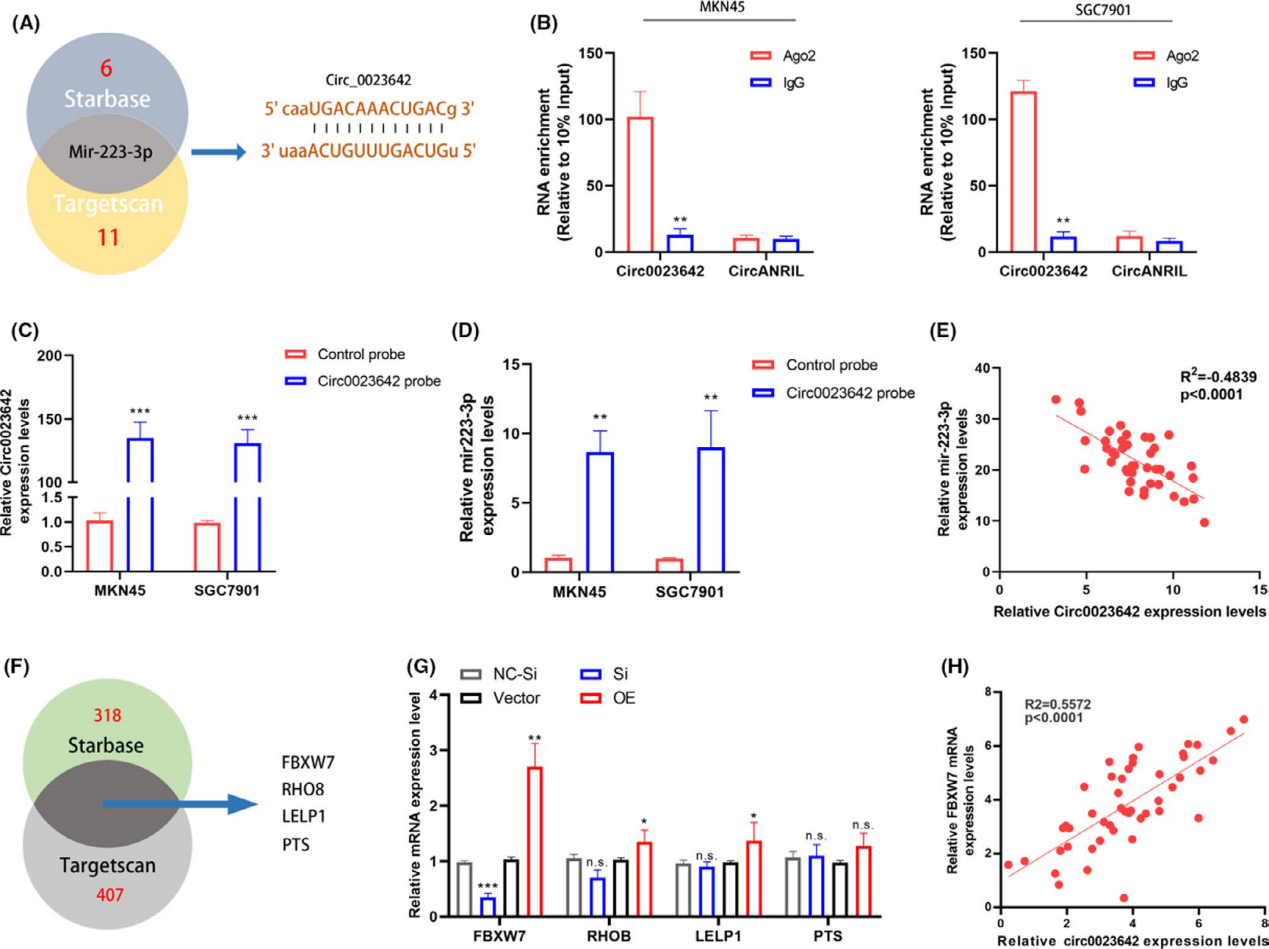
hsa_circ_0023642 is a sponge of miR‐223a‐3p. A, The Venn diagram shows the intersection of miRNA lists. B, RIP experiments were performed using an antibody against AGO2 with extracts from MKN‐45 and SCG7901 cells. C, qRT‐PCR results showed that hsa_circ_0023642 could be specifically enriched by hsa_circ_0023642 probe. D, The relative expression levels of miR‐223‐3p was detected by qRT‐PCR inMKN‐45 and SCG7901 cell lysates. E, The expression of hsa_circ_0023642 and miR‐223‐3p are inversely correlated in GC tissues. F, The Venn diagram shows the intersection of mRNAs lists between StarBase and Targetscan. G, The mRNA levels of FBXW7, RHOB, LELP1 and PTS in MKN45 cells with knockdown or overexpression of hsa_circ_0023642 was determined by qRT‐PCR. H, Expression of FBXW7 and hsa_circ_0023642 are positively correlated in GC tissues (*R* ^2^ = 0.5572, *P* < .0001, n = 46)

It has been known that circRNAs play many important roles, one of which is acting as miRNA sponge to regulate gene expression. To explore miRNA sponge ability of hsa_circ_0023642 in GC cells, one miRNA (miR‐223‐3p) was selected from the prediction results through bioinformation analysis database (StarBase and Targetscan) (Figure 5A). Next, we conducted an RIP assay with an antibody against AGO2 in GC cells. The results showed that hsa_circ_0023642 but not the negative control circANRIL was significantly enriched using the anti‐AGO2 antibody (Figure 5B). Subsequently, we designed a hsa_circ_0023642‐specific probe labeled with biotin to perform pull‐down assay after overexpressed hsa_circ_0023642 in GC cell lines. As a positive control, the level of hsa_circ_0023642 was remarkably higher in hsa_circ_0023642 targeted probe group than oligo probe group (Figure 5C). And we found that miR‐223‐3p was copiously pulled down in MKN45 and SGC7901 cells (Figure 5D). Furthermore, a significant inverse correlation occurred between hsa_circ_0023642 and miR‐223‐3p in GC tissues (*R* ^2^ = −0.4839, *P* < .001; Figure 5E). These results suggested that hsa_circ_0023642 could directly target miR‐223‐3p and function as a sponge for miR‐223‐3p in GC cells. We then used the Starbase and Targetscan databases to predict the target genes that mir‐223‐3p might act on. And FBXW7, RHOB, LELP1, and PTS were found to have higher scores in both two databases (Figure 5F). Subsequently, we used PCR for analysis and found that only FBXW7 was consistent with the change of hsa_circ_0023642 after knockingdown or overexpression of hsa_circ_0023642 (Figure 5G), and the expression level of FBXW7 in the tissue samples was also highly correlated with hsa_circ_0023642 (Figure 5H).

## 4 DISCUSSION

Given the current developments in high‐throughput sequencing technology, the role of circRNA in carcinogenesis and cancer development has been widely explored.^11^ Functional studies on circRNAs continue to expand and deepen in a variety of physiological and pathological processes.^12^ In this study, we analyzed human circRNA microarray data and found for the first time that hsa_circ_0023642 is expressed in higher levels in GC than in adjacent non‐tumor tissues. It has been reported in the literature that silencing circular RNA UVRAG (also known as Hsa_circ_0023642) inhibited bladder cancer growth and metastasis by targeting the miR‐223/FGFR2 axis.^13^, ^14^ However, compared with the study in bladder cancer, the level of expression and function of hsa_circ_0023642 in GC development are still largely unknown, and the target genes we have done were different. We found the phenomenon that the clinicopathological features of these patients indicate that hsa_circ_0023642 is closely related to the clinical malignant progression of GC. We found that hsa_circ_0023642 is closely related to lymph node metastasis and prognosis. The higher the expression level of hsa_circ_0023642 is, the worse the malignant degree of gastric adenocarcinoma will be. The fact that circRNA is abundantly found in tissue or blood makes circRNA a valuable diagnostic biomarker for assessing the progression and prognosis of GC.^15^

Our research is not the first to show that circRNA is relevant in the malignant progression of GC. Chen et al found that circ_0000190 is decreased in GC tissues and serum and that its expression level is correlated with tumor diameter, lymph node metastasis, distant metastasis, TNM stage, and CA19‐9 level.^16^ Huang found that circ_0000745 is downregulated in GC tissue and plasma samples. Its expression in GC is related to the degree of tumor differentiation, whereas its expression in plasma is related to tumor TNM staging.^17^ In other tumors, circRNA also shows tumor growth and migration and related functions. For example, Hsa_circ_0067934 is upregulated in esophageal squamous cell carcinoma (ESCC), and the higher the expression level is, the worse the degree of tumor differentiation will be.^18^ In their study, hsa_circ_0067934 promotes the proliferation and migration of ESCC cells. Xu et al^19^ demonstrated that high ciRS‐7 (Cdr1as) expression in hepatocellular carcinoma cancer tissue can shorten the median time for relapse in patients.

CircRNA is not only a by‐product of mis‐splicing or splicing errors as it can also act between epithelial and mesenchymal transitions. CircRNAs are enriched in and function well at miRNA binding sites, and they work in various ways, including their action as miRNA sponges, RBP sponges, and mRNA modulators.^20^, ^21^ Moreover, hsa_circ_0000673 potentially acts as a sponge for the oncogene R‐532‐5p, which upregulates RUNX3, p21, and Bim expression and thus inhibits GC proliferation and invasion.^22^ Circ‐ZFR inhibits GC cell proliferation and promotes apoptosis through the sponge miR‐130a/ miR‐107 and regulation of PTEN.^23^

In this study, we identified a novel circRNA, hsa_circ_0023642, which was upregulated in GC tissues and related with the malignant progression. Mechanistically, hsa_circ_0023642 was demonstrated could as a miRNA sponge to inhibit the miR‐233‐3p. The target genes of miR‐233‐3p, FBXW7, was significantly regulated by the expression of hsa_circ_0023642. Thus, we identified that hsa_circ_0023642 promoted the malignant prognosis of GC by hsa_circ_0023642/miR‐233‐3p/FBXW7 axis. These findings suggest hsa_circ_0023642 could play a significant role and a critical biomarker in GC.

## 5 CONCLUSION

In summary, hsa_circ_0023642 was expressed higher in GC compared with the non‐GC. Hsa_circ_0023642 correlated with diagnosis and prognosis of GC might be used as a potential biomarker for early diagnosis and prognosis of patients with GC.
